# The Role of Matrix Metalloproteinase-2 (MMP2) in Colorectal Cancer Progression: Correlation With Clinicopathological Features and Impact on Cellular Processes

**DOI:** 10.7759/cureus.61941

**Published:** 2024-06-08

**Authors:** Qais Ibraheem

**Affiliations:** 1 Department of Anatomy, Biology and Histology, College of Medicine, University of Duhok, Duhok, IRQ

**Keywords:** apoptosis, proliferation, cell invasion and migration, matrix metalloproteinase-2, colorectal cancer

## Abstract

Background

Colorectal cancer (CRC) is a prevalent and deadly disease characterized by significant molecular complexity. Matrix metalloproteinase-2 (MMP2) has been implicated in cancer progression due to its role in extracellular matrix degradation, yet comprehensive studies linking MMP2 expression to CRC progression and its molecular mechanisms remain needed.

Methodology

This study involved 90 CRC patients, with tumor and adjacent normal tissues analyzed via immunohistochemistry (IHC) to assess MMP2 expression. The human CRC cell line SW480 was treated with an MMP2 inhibitor, ARP100, and evaluated for changes in cell migration, invasion, proliferation, and apoptosis using various assays, including MTT, wound-healing, transwell, caspase activity, and western blot analysis.

Results

High MMP2 expression was significantly associated with advanced tumor stages, lymph node involvement, and metastasis in CRC patients. Compared to normal tissues, MMP2 expression was markedly higher in cancerous tissues. Inhibition of MMP2 in SW480 cells resulted in reduced migration, invasion, and proliferation, and induced apoptosis, evidenced by increased caspase 3 and 9 activities and higher levels of cleaved caspase proteins.

Conclusion

Elevated MMP2 expression is correlated with advanced CRC and aggressive tumor characteristics. MMP2 inhibition can suppress CRC cell invasiveness, migration, and proliferation while promoting apoptosis, suggesting its potential as a therapeutic target in CRC treatment.

## Introduction

Colorectal cancer (CRC) is a major global health concern, representing a significant cause of cancer-related morbidity and mortality worldwide. Despite advancements in diagnosis and treatment modalities, CRC remains a formidable challenge due to its heterogeneity and complex molecular landscape [1]. The identification of key molecular players involved in CRC progression is crucial for developing targeted therapeutic strategies and improving patient outcomes. Matrix metalloproteinases (MMPs) constitute a family of zinc-dependent endopeptidases that play pivotal roles in extracellular matrix (ECM) remodeling, tissue homeostasis, and various pathological processes including cancer progression and metastasis [2]. Among MMPs, MMP2 (gelatinase A) has emerged as a key mediator in cancer biology due to its ability to degrade type IV collagen, a major component of the basement membrane, thereby facilitating cancer cell invasion and metastasis [3]. Understanding the role of MMP2 in CRC pathogenesis is of paramount importance as it can provide insights into disease aggressiveness, prognosis, and potential therapeutic targets. Several studies have implicated MMP-2 dysregulation in CRC progression, correlating its expression levels with clinicopathological features such as tumor stage, metastasis, and patient survival [4]. However, comprehensive investigations integrating MMP2 expression with multiple cellular processes and clinical parameters are warranted to elucidate its multifaceted role in CRC. The primary aim of this study is to investigate the role of MMP2 in CRC progression by correlating its expression levels with clinicopathological features and exploring its impact on key cellular processes including migration, invasion, proliferation, and apoptosis in CRC cells.

## Materials and methods

Sample collection

Following the acquisition of informed consent and approval from the Research Ethics Committee of the College of Medicine at the University of Duhok, a cohort of 90 patients diagnosed with CRC was included in this study. The study included patients who were over 16 years old, had a histopathological diagnosis of CRC, and were scheduled for surgery, chemotherapy, or radiotherapy; their histopathological samples were used for immunohistochemical analysis. Patients were excluded if they were unwilling to participate, had incomplete examination data (such as demographics, CRC location, staging, and histopathology characteristics), had unreadable immunohistochemical staining, or had autoimmune and inflammatory diseases. Post-surgical resection, the tissue specimens were preserved in 10% neutral buffered formalin and subsequently embedded in paraffin blocks. Four-micrometer tissue sections were then stained with hematoxylin and eosin (H&E) for further histopathological analysis. Additionally, 40 matched normal adjacent tissues were collected from the aforementioned 90 patients.

Immunohistochemistry (IHC)

All the IHC reagents, including the SP Detection Kit, IHC Biotin Block Kit, and DAB Visualization Kit were obtained from Dako, Denmark. The experiments followed the manufacturers' instructions. Paraffin sections (4 mm thick) from 90 CRC samples and 40 para-cancerous matched normal tissues were deparaffinized in 100% xylene and rehydrated through a graded ethanol series and water according to standard protocols. Heat-induced antigen retrieval was carried out in a 10 mM citrate buffer for two minutes at 100°C. Non-specific antigens were blocked using a peroxidase-blocking reagent containing 3% hydrogen peroxide and serum. The sections were then incubated overnight with a mouse anti-human MMP2 antibody (1:200; Abcam, USA). After washing, the sections were incubated with a biotin-labeled goat anti-mouse IgG antibody (Abcam, USA) for 10 minutes at room temperature, followed by incubation with streptavidin-conjugated horseradish peroxidase (HRP) (Dako, Denmark). Visualization was achieved using diaminobenzidine (DAB), and the sections were counterstained with hematoxylin, mounted in neutral gum, and analyzed with a bright field microscope (Olympus, Japan).

IHC scoring

Expression of protein was assessed by two independent pathologists using a semi-quantitative scoring system that measured both the staining intensity and the distribution of positive cells. The intensity of the positive reaction was graded on a scale of negative (0), weak (1), moderate (2), and strong (3). The percentage of positively stained cells was categorized as follows: 0-5% (0), 6-25% (1), 26-51% (2), 51-76% (3), and 76-100% (4). The staining index score, ranging from 0 to 12, was calculated by multiplying the staining intensity score by the proportion of positive cells. For statistical analysis, a final staining score of 0-6 indicated low protein expression and a score of 6-12 was considered to be a high expression [4].

Cell culture and treatment

Human CRC cell line SW480 was acquired from the American Type Culture Collection. These cells were grown in RPMI 1640 medium supplemented with 10% fetal bovine serum (Sigma Aldrich, Germany), 100 U/mL penicillin (Sigma Aldrich, Germany), and 100 mg/mL streptomycin (Sigma Aldrich, Germany). The cell cultures were kept in a humidified incubator at 37℃ with 5% CO2. For cell treatment, cells were initially cultured in 60 mm dishes with complete media until reaching 50% confluence as previously described [5]. Following this, the media was replaced with serum-free media, and the cells were left for 24 hours. Subsequently, the dishes were further incubated with 5 μM of the MMP2 inhibitor ARP100 (MMP-2 inhibitor from Santa Cruz Biotechnology, Dallas, USA), dissolved in dimethyl sulfoxide (DMSO) at the specified concentration as needed for the experiments.

MTT assay

To evaluate how drug-mediated MMP-2 suppression impacts CRC cell viability, an MTT colorimetric assay was performed on SW480 cells three days post-treatment. The cells were seeded into individual wells of 96-well plates, with each well containing 2000 cells. The plates were then maintained at 37°C for one to three days. Daily, 20 μl of a 5 mg/mL solution of 3-(4,5-dimethylthiazol-2-yl)-2,5-diphenyl-tetrazolium bromide (MTT) from Thermo Fisher Scientific (Waltham, USA) was added to each well and incubated for four hours. Following this, a solution of acidic isopropanol (comprising 10% sodium dodecyl sulfate (SDS), 5% isopropanol, and 0.01 M hydrochloric acid (HCl)) at 100 μl per well was added and left to incubate overnight at 37°C. The optical density (OD) was then measured at 570 nm using a microplate reader from Bio-Rad (Hercules, USA).

Wound-healing assay

The migration of SW480 cells treated with either a drug or DMSO (control) was evaluated using a wound-healing assay. Cells were seeded at a density of 1.5×10^6 ^cells/well in six-well plates and allowed to grow overnight until they reached 90% confluence. A straight scratch was created using a sterile pipette tip, and any detached cells were gently washed off with phosphate-buffered saline (PBS) three times. The cells were then cultured in the medium for an additional 24 hours. The cell movement was monitored and photographed using a digital camera from Leica (Germany) at both 0 hours and 24 hours.

Transwell migration and invasion assay

The migration and invasion assays were carried out using a 24-well transwell system with 8 μm pore size filters from Corning (USA). For the migration assay, 5 × 10^4^ cells were suspended in 300 µl of serum-free medium and placed in the upper chamber, while the lower chamber was filled with 800 µl of medium containing 10% fetal bovine serum (FBS). After a 48-hour incubation, the chamber was fixed with 4% paraformaldehyde and stained with 0.5% crystal violet. Cells in the upper chamber were then wiped off with a cotton swab, and cells in four randomly selected microscopic fields were counted and photographed. For the invasion assay, the same procedure was followed, except that 1 × 10^5^ cells were seeded into the upper chamber precoated with matrigel from BD Biosciences (USA). Each experiment was conducted in triplicate to ensure consistency.

Caspase activity assay

Caspase 3 and 9 activity in treated cells was assessed using Caspase 3 and 9 Activity Assay Kits from Elabscience Biotechnology Inc. (Houston, USA) following the manufacturer's protocols. In brief, 1x10^5^ cells treated with inhibitor and DMSO as control were cultured in 96-well plates and incubated for 24 hours. The cells were then detached, and centrifuged at 10,000 g for five minutes, and 55 μl of working solution buffer containing 50 μl of 2× reaction working solution and 5 μl of Ac-DEVD-pNA was added to the cells. The cell plate was placed on an oscillating shaker for 30 minutes, and the optical density was measured using a microplate reader from Bio-Rad.

Western blot

Cells were treated with Laemmli buffer from Thermo Fisher Scientific (USA) to lyse them, followed by centrifugation at 12,000 g for 10 minutes to separate the supernatant-containing proteins. Equal amounts of protein from each experimental group were loaded onto sodium dodecyl sulfate-polyacrylamide gel electrophoresis (SDS-PAGE). The proteins were transferred onto a polyvinylidene fluoride (PVDF) membrane from Millipore (MA, USA) and incubated overnight at 4°C with the mouse anti-human MMP2 antibody (1:200). After washing the membranes with PBS, they were then exposed to a species-specific secondary antibody linked to HRP for two hours at room temperature. Finally, visualization of the proteins on the membranes was achieved using an enhanced chemiluminescence kit from Millipore (MA, USA).

RNA extraction, complementary DNA (cDNA) synthesis, and quantitative polymerase chain reaction (PCR)

Cells underwent RNA extraction using the RNeasy RNA Isolation Kit from Germantown (MD, USA), after which reverse transcription into cDNA was performed using the RevertAid H Minus First Strand cDNA Synthesis Kit from Thermo Fisher Scientific. Quantitative PCR was carried out in triplicate using SYBR Green PCR Master Mix from Applied Biosystems (Warrington, UK). The quantitative data obtained were normalized to the GAPDH internal control, and data analysis was conducted using the conventional ΔΔCT method. The primers used in this study were designed and verified using BLAST software (National Center for Biotechnology Information, Bethesda, USA). The sequences of the primers are as follows: MMP2; (F) 5’ TTGGCAGTGCAATACCTGAA 3’ and (R)5’ GAGTCCGTCCTTACCGTCAA 3’, while for GAPDH; (F) 5’ GTCAAGGCTGAGAACGGGAA3’ and 5’CAGCCACGAACACGATGAAC3’.

Statistical analysis

All experiments conducted in vitro were repeated a minimum of two times to mitigate inconsistencies. Data comparisons among study groups were carried out using GraphPad Prism 9.0 software (GraphPad Software, San Diego, CA) through one-way analysis of variance (ANOVA) and t-tests. For immunohistochemical experiments, statistical analyses were conducted using the Statistical Package for the Social Sciences (IBM SPSS Statistics for Windows, IBM Corp., Version 20.0, Armonk, NY). Associations between protein expression and clinicopathological factors, as well as between cancer and normal tissues, were explored using Fisher's exact or Chi-square tests. Statistical significance was determined for all analyses with a p-value less than 0.05.

## Results

Demographic information on CRC patients

The study comprised predominantly older patients, with 68.9% being over 50 years of age. The gender distribution was relatively balanced, with 52.2% male and 47.8% female. Adenocarcinoma was the most common histological type, present in 90% of the cases, while mucinous carcinoma accounted for 10%. The majority of tumors were larger than 5 cm (73.3%) and moderately differentiated (Grade 2, 66.7%). Tumor stages were mostly advanced, with 35.6% at Stage 2 and 36.7% at Stage 3. Regarding tumor invasion (T), most were T3 (55.6%). Lymph node involvement was absent in 60% of patients (N0), and distant metastasis was uncommon, with 88.9% having no distant metastasis (M0). Vascular invasion was present in 64.4% of the cases, and perineural infiltration was observed in 53.3% of the patients (Table 1).

**Table 1:** Clinicopathological features of the patients T: tumor depth; N: lymph node involvement; M: metastasis

Variables		No.	%
Age (years)	<=50	28	31.1
	>50	62	68.9
Sex	Male	47	52.2
	Female	43	47.8
Histological type	Adeno	81	90.0
	Mucinous	9	10.0
Size of tumor (cm)	<=5	24	26.7
	>5	66	73.3
Grade	1	10	11.1
	2	60	66.7
	3	20	22.2
Stage	1	19	21.1
	2	32	35.6
	3	33	36.7
	4	6	6.7
T	1	6	6.7
	2	23	25.6
	3	50	55.6
	4	11	12.2
N	0	54	60.0
	1	21	23.3
	2	15	16.7
M	1	80	88.9
	2	10	11.1
Vascular invasion	Negative	32	35.6
	Positive	58	64.4
Perineural infiltration	Negative	42	46.7
	Positive	48	53.3
Total		40	100.0

MMP2 expression associated with the advanced stage of CRC patients

The analysis of the correlation between MMP2 expression and clinicopathological features reveals several significant associations. There are strong correlations with tumor stage, lymph node involvement, and metastasis. High MMP2 expression is significantly associated with more advanced stages (P<0.001), greater lymph node involvement (P=0.003), and higher rates of metastasis (P=0.044). Vascular invasion and perineural infiltration are also significantly higher in the high MMP2 group (P<0.001 and P=0.035, respectively). However, Age, sex distribution, tumor size, and histological type of cancer do not significantly differ between low and high MMP2 expression groups. This suggests that high MMP2 expression is correlated with more aggressive and advanced tumor characteristics (Table 2).

**Table 2:** Correlation between MMP2 expression and clinicopathological features of CRC patients T: tumor depth; N: lymph node involvement; M: metastasis; MMP2: matrix metalloproteinase-2; CRC: colorectal cancer * Based on Chi-square test, or (if not applicable) Fisher’s exact test

Variables		MMP-2				
		Low expression		High expression		P-value*
		No.	%	No.	%	
Age (years)	<=50	15	33.3	13	28.9	0.649
	>50	30	66.7	32	71.1	
Sex	Male	22	48.9	25	55.6	0.527
	Female	23	51.1	20	44.4	
Histological type	Adeno	41	91.1	40	88.9	1.000
	Mucinous	4	8.9	5	11.1	
Size of tumor (cm)	<=5	8	17.8	16	35.6	0.057
	>5	37	82.2	29	64.4	
Grade	1	6	13.3	4	8.9	0.122
	2	33	73.3	27	60.0	
	3	6	13.3	14	31.1	
Stage	1	16	35.6	3	6.7	<0.001
	2	20	44.4	12	26.7	
	3	7	15.6	26	57.8	
	4	2	4.4	4	8.9	
T	1	4	8.9	2	4.4	0.104
	2	14	31.1	9	20.0	
	3	25	55.6	25	55.6	
	4	2	4.4	9	20.0	
N	0	35	77.8	19	42.2	0.003
	1	6	13.3	15	33.3	
	2	4	8.9	11	24.4	
M	1	43	95.6	37	82.2	0.044
	2	2	4.4	8	17.8	
Vascular invasion	Negative	24	53.3	8	17.8	<0.001
	Positive	21	46.7	37	82.2	
Perineural infiltration	Negative	26	57.8	16	35.6	0.035
	Positive	19	42.2	29	64.4	
Total		45	100.0	45	100.0	

MMP2 expression elevated in cancerous tissues compared to adjacent normal tissues in CRC

**Figure 1:**
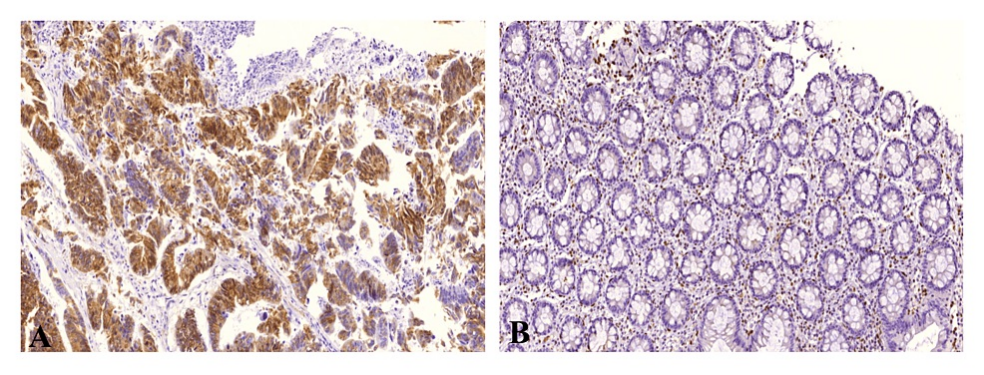
Differences in the expression of MMP2 level between cancerous and normal adjacent tissues The representative image showed elevated MMP2 expression levels in (A) cancerous tissues compared to those in (B) normal tissues (20x). MMP2: matrix metalloproteinase-2

The analysis of MMP2 expression between cancer tissues and adjacent normal tissues reveals a significant difference (P<0.001) (Table 3). Among the cancer cases, MMP2 expression is evenly split, with 50% (45 cases) showing low expression and 50% (45 cases) showing high expression. In contrast, the adjacent normal tissues predominantly exhibit low MMP2 expression, with 92.5% (37 controls) showing low expression and only 7.5% (three controls) showing high expression (Figure 1). This marked difference suggests that high MMP2 expression is significantly associated with cancerous tissues, highlighting its potential role in tumorigenesis.

**Table 3:** Differences in MMP2 expression between cancerous and cancerous-adjacent normal tissues * Based on Chi-square test, or (if not applicable) Fisher’s exact test; MMP2: matrix metalloproteinase-2

MMP-2	Cases		Controls		Total		P-value*
	No.	%	No.	%	No.	%	
Low expression	45	50.0	37	92.5	82	63.1	<0.001
High expression	45	50.0	3	7.5	48	36.9	
Total	90	100.0	40	100.0	130	100.0	

Inhibition of MMP2-suppressed SW480 cell invasion and migration

**Figure 2:**
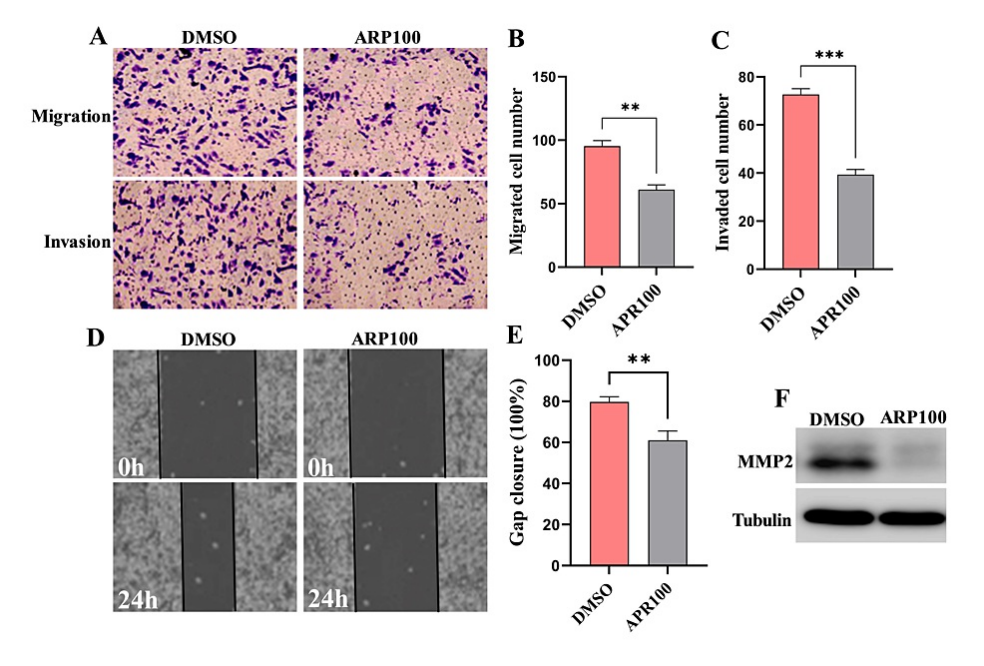
MMP2 inhibition suppresses migration and invasion of CRC cells MMP2 was inhibited in SW480 cells by treating with 5 μM of the MMP2 inhibitor ARP100 for 24h, while control cells were treated with DMSO. (A-C) Transwell migration and invasion assays (magnification: 100×) demonstrated that MMP2 inhibition significantly reduced the migratory and invasive capabilities of SW480 cells. (D and E) Wound healing assays (scale: 100 µm) showed that MMP2 inhibition markedly decreased gap closure in SW480 cells. Data are presented as mean ± standard error (**P≤0.002; ***P<0.0005). (F) Western blot analysis confirmed MMP2 inhibition. Results are representative of three independent experiments. MMP2: matrix metalloproteinase-2; CRC: colorectal cancer; DMSO: dimethyl sulfoxide

To determine MMP2's functional role in metastasis, we evaluated its effects on the migration and invasion of SW480 cells using Transwell and wound healing assays. The results from the Transwell assay showed a notable decrease in both migratory and invasive abilities (Figures 2A-2C) of APR100-SW480 treated cells (P≤0.002 and P≤0.0005, respectively) compared to the control group. Wound healing assay results indicated that MMP2 inhibition significantly slowed gap closure in SW480 cells (Figures 2D-2E) compared to the control group (P=0.002). Western blot analysis confirmed MMP2 inhibiting, demonstrating significant changes in protein expression post-treatment (Figure 2F). Overall, these findings provide evidence linking the MMP2 protein to the increased migratory and invasive characteristics of SW480 cells.

Inhibition of MMP2-suppressed SW480 cell proliferation

**Figure 3:**
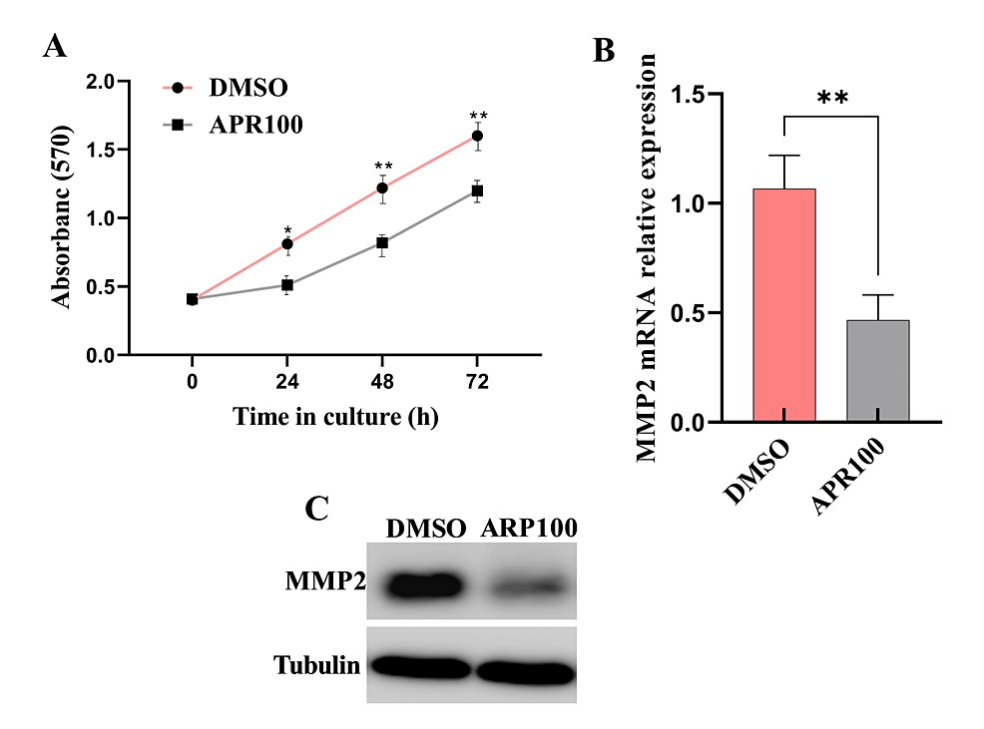
MMP2 expression contributes to the proliferation of colorectal cancer cells MMP2 was inhibited in SW480 cells by treating with 5 μM of the MMP2 inhibitor ARP100, while control cells were treated with DMSO. Cell proliferation was assessed using the MTT assay, with absorbance readings taken at 570 nm. (A) APR100 inhibitor significantly inhibited SW480 cell proliferation. (B and C) Transfection efficiency was validated through RT-PCR and western blot analysis. Data are presented as mean ± standard error (*P<0.02 and **P≤0.009). Results from three representative experiments are depicted. MMP2: matrix metalloproteinase-2; DMSO: dimethyl sulfoxide; MTT: 3-(4,5-dimethylthiazol-2-yl)-2,5 diphenyl tetrazolium bromide; RT-PCR: reverse transcription-polymerase chain reaction

We proceeded to examine MMP2's functional impact on CRC cell proliferation. To do this, we inhibited MMP2 expression in SW480 cells. Subsequently, we conducted the MTT assay to assess cell proliferation in the treated cells. According to the MTT results, MMP2 inhibiting notably hindered SW480 cell proliferation at days 1, 2, and 3 compared to the control group (P<0.02; P≤0.009 and P≤0.009) (Figure 3A). We verified transfection efficiency through real-time PCR and western blot analyses (Figures 3B-3C). Together, these results suggest that MMP2 supports the proliferative capacity of CRC cells.

Inhibition of MMP2-induced SW480 cells apoptosis

**Figure 4:**
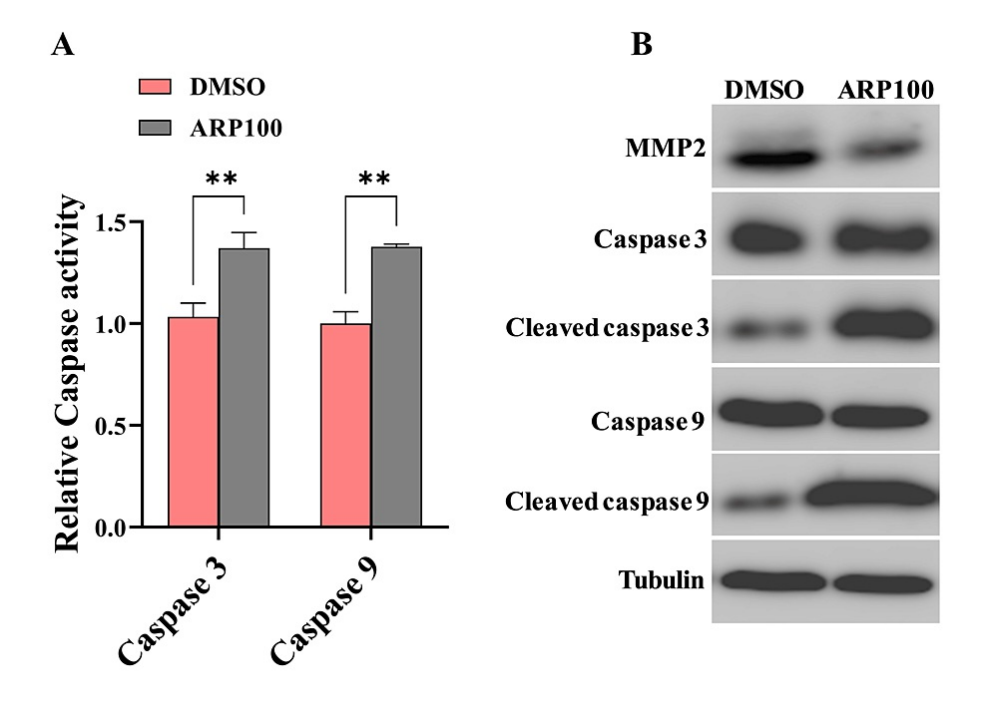
MMP2 inhibition induces apoptosis in SW480 cells line Caspase 3 and 9 activities in SW480 treated cells were quantified using spectrophotometry. The activity of caspases in control cells (treated with DMSO) was set as 1, and the activity values of the experimental group were standardized relative to this. (A) SW480 cells treated with inhibitor exhibited a significant elevation in both caspase 3 and caspase 9 activities. (B) Western blot analysis was performed to detect the expression levels of apoptosis-related proteins. Bar graphs depict the standard errors of the mean from three experiments (**P≤0.005 or P=0.001). MMP2: matrix metalloproteinase-2; DMSO: dimethyl sulfoxide

Since reducing MMP2 levels hindered SW480 cell proliferation, we conducted caspase 3 and 9 activity assays to explore MMP2's role in SW480 cell apoptosis. As previously mentioned, SW480 cells were treated with 5 μM of the MMP2 inhibitor ARP100 and DMSO as a control for 24h. The treated cells were then analyzed for caspase activity. The results revealed that MMP2 inhibiting significantly reduced caspase 3 and caspase 9 activity in APR100 treated cells compared to the control group (P≤0.005; P=0.001, respectively) (Figure 4A). Furthermore, cell lysates collected 24 hours post-treatment underwent western blot analysis to detect apoptosis-associated protein levels. In MMP2-inhibiting cells, the expression levels of proapoptotic proteins (cleaved caspase 3 and cleaved caspase 9) significantly increased compared to control cells. However, no significant changes were observed in caspase 3 or caspase 9 expression across the groups (Figure 4B). This data suggests that MMP2 may play a role in regulating the apoptotic process in CRC cells.

## Discussion

MMP2 protein levels are generally low or undetectable in normal tissues and cells. However, they are frequently overexpressed during particular cellular processes such as wound healing, tissue remodeling, and embryonic development [6]. MMP2 can degrade most components of the ECM and basement membrane, facilitating the invasion and spread of tumor cells [7]. Consequently, it plays a role in the progression and metastasis of various tumors. The positive expression of MMP2 has been shown to be associated with advanced-stage retinoblastoma [8], oral cancers [9] ovarian epithelial cancer [10], and bladder cancer [11].

In this study, immunohistochemical analysis revealed that high expression of MMP2 was significantly correlated with tumor stage, lymph node metastasis, vascular invasion, and perineural invasion. Additionally, we found that MMP2 expression was remarkably increased in cancer tissues, compared to normal adjacent tissue. These results were aligned with the findings of Lipari et al. and Jeffery et al. [12,13], indicating that elevated MMP2 levels contribute to disease progression in CRC patients and have a detrimental effect on CRC pathogenesis.

It is well established that MMP2 plays a crucial role in different steps of cancer progression including invasion, migration, proliferation, and apoptosis [14]. To gain a deeper understanding of how MMP2 promotes tumor cell growth and invasion, we inhibited MMP2 expression in the SW480 cell line using an APR100 inhibitor. Consistent with previous studies [15-18], we observed that inhibiting MMP2 expression significantly decreased cell proliferation and invasiveness in vitro. These findings indicate that MMP2 plays a promoting role in the development and progression of CRCs. Furthermore, our findings revealed that caspase activity, as well as the levels of cleaved caspase 3 and 9, was significantly elevated in MMP2-suppressed SW480 cells compared to control cells. The findings of MMP2's involvement in the regulation of apoptosis in this study are consistent with those reported by [19,20].

Study limitation

One limitation of this study is the relatively small sample size of 90 CRC patients, which may not be representative of the broader population and could affect the robustness and generalizability of the findings. Additionally, the study's cross-sectional design limits the ability to draw causal inferences between MMP2 expression and CRC progression. The reliance on IHC for assessing MMP2 expression, while useful, may not fully capture the dynamic and multifaceted role of MMP2 in tumor biology. Furthermore, the exclusion of patients with autoimmune, inflammatory, or fibrotic diseases may overlook important interactions between these conditions and MMP2 expression in CRC. Finally, the use of a single CRC cell line (SW480) for in vitro experiments may not fully represent the heterogeneity of CRC, necessitating further studies using multiple cell lines and in vivo models to validate these findings and explore the therapeutic potential of MMP2 inhibition.

## Conclusions

In summary, our study showed that MMP2 expression was significantly elevated in CRC and its expression was associated with the advanced stage of CRC patients. Additionally, our findings indicated that inhibiting MMP2 could diminish cell invasion, migration, and proliferation, as well as induce apoptosis.
